# Evolution of Prognosis and Management in Curative Treatment of Locally Recurrent Rectal Cancer: A Comprehensive Analysis from 2000 to 2023

**DOI:** 10.1245/s10434-026-20398-6

**Published:** 2026-08-20

**Authors:** Fleur E. C. Vande Kerckhove, Davy M. J. Creemers, Evi Banken, Floor Piqeur, Stijn H. J. Ketelaers, R. R. J. Coebergh van den Braak, Jip Tolenaar, Robert-Jan Schipper, Grard A. P. Nieuwenhuijzen, Wikke Setz-Pels, Thijs R. van Oudheusden, Alette W. Daniëls-Gooszen, Stefan G. van Ravensteijn, Irene E. G. van Hellemond, An-Sofie E. Verrijssen, Heike M. U. Peulen, Johanne G. Bloemen, Joost Nederend, Harm J. T. Rutten, Jacobus W. A. Burger

**Affiliations:** 1 Department of Surgery, Catharina Hospital Eindhoven, Eindhoven, The Netherlands; 2 GROW Research Institute for Oncology and Reproduction, Maastricht University, Maastricht, The Netherlands; 3 Department of Medical Oncology, Catharina Hospital Eindhoven, Eindhoven, The Netherlands; 4 Department of Radiation Oncology, Catharina Hospital Eindhoven, Eindhoven, The Netherlands; 5 Department of Radiology, Catharina Hospital Eindhoven, Eindhoven, The Netherlands

**Keywords:** Locally recurrent rectal cancer, Survival, Neoadjuvant treatment, Surgery

## Abstract

**Background:**

Despite advances in diagnosis and treatment, locally recurrent rectal cancer (LRRC) remains a complex condition associated with a poor prognosis. This study aimed to evaluate long-term trends in the management of and oncological outcomes for patients with LRRC treated in a Dutch tertiary referral center.

**Methods:**

All consecutive patients with a confirmed diagnosis of a first LRRC undergoing curative-intent surgical resection at our referral center between 2000 and 2023 were retrospectively analyzed. Primary outcomes were trends in overall survival (OS), disease-free survival (DFS), clear (R0) resection rate, and postoperative complication rates. Cumulative sum (CUSUM) analysis was used to visualize changes in these outcomes over time. Secondary endpoints included temporal changes in prognostic factors associated with improved OS and DFS.

**Results:**

A total of 560 patients undergoing curative-intent surgical resection were included. Over time, patients presented with more comorbidities (*p* < 0.001), more multifocal disease (*p* < 0.001), and synchronous metastasis (*p* < 0.001). The R0 resection rate improved from 59% to 78% over the study period (*p* < 0.001). CUSUM analyses demonstrated an upward trend in OS and DFS, indicating a cumulative improvement in survival relative to the reference survival probability. In univariable Cox regression, the most recent time period (2018–2023) was significantly associated with improved OS (hazard ratio 0.75; 95% confidence interval 0.58–0.97; *p *= 0.030).

**Conclusions:**

Over the past two decades, the outcomes of patients undergoing curative-intent treatment for LRRC have steadily improved. This may reflect advances in diagnostics, neoadjuvant therapy, surgical technique and perioperative care, centralization of care, and patient selection.

**Supplementary Information:**

The online version contains supplementary material available at https://doi.org/10.1245/s10434-026-20398-6.

Despite substantial advancements in the detection, diagnostic evaluation, and multimodal management of locally recurrent rectal cancer (LRRC), its treatment remains challenging. Currently, only 35–45% of patients are eligible for curative-intent treatment. The remaining patients are precluded from curative treatment because of distant metastases, irresectability, poor clinical condition, or frailty.^1–3^

Widespread implementation of and advancements in diagnostic modalities, such as high-resolution magnetic resonance imaging (MRI) with diffusion-weighted imaging, computed tomography (CT), and positron emission tomography (PET), have progressively enhanced the accuracy of LRRC detection. Efforts to centralize care in tertiary referral centers, initiated in the 1990s and progressively implemented throughout the 2000s and 2010s, have increased patient concentration and facilitated the development of dedicated multidisciplinary teams (MDTs). Collectively, these advancements have potentially contributed to a reduction in futile surgical resections and to improved oncological outcomes.

Surgical resection with a clear margin (R0) remains the cornerstone of curative treatment for LRRC.^4^ To induce preoperative downstaging and increase the likelihood of R0, neoadjuvant chemoradiotherapy (CRT) has been widely adopted.^5–7^ In patients who have previously received radiotherapy for the primary tumor, chemo-reirradiation is considered the standard of care in the Dutch national guidelines.^5^ Its use is considered in the European Society for Medical Oncology and Beyond-Total Mesorectal Excision guidelines.^6,7^ However, this approach remains controversial because of concerns regarding cumulative toxicity. Likewise, the role of neoadjuvant induction chemotherapy (ICT) for LRRC remains controversial, as potential benefits, such as improved oncological outcomes, have not been conclusively demonstrated in a trial setting. Although ICT is not considered standard of care in the Netherlands, it is adopted in many other countries worldwide. From 2020 onwards, the value of ICT is being studied in an ongoing randomized controlled trial running in the Netherlands, Norway, Sweden, Belgium, and Portugal.^8^ In parallel, significant improvements in perioperative care have made it possible to safely conduct more extensive surgical procedures with acceptable morbidity and mortality rates.^9^ In patients undergoing less complex procedures, morbidity and mortality are likely to have improved.^10,11^

In light of the aforementioned evolving multimodal management of LRRC, the aim of the present study was to evaluate global temporal developments with regard to both short-term and long-term outcomes over the past two decades in a Dutch tertiary referral center.

## Materials and Methods

### Patient Selection

All patients with a confirmed diagnosis of LRRC who received treatment at Catharina Hospital Eindhoven, a national referral center, were included in a prospectively maintained database. All consecutive patients undergoing a curative-intent surgical resection between January 2000 and December 2023 were retrospectively identified and reviewed. All patients were discussed in an MDT to achieve consensus on treatment intention, neoadjuvant therapy, response assessment, and subsequent surgical therapy. MDTs consisted of surgical oncologists, medical oncologists, gastroenterologists, radiation oncologists, radiologists, and pathologists. LRRC was defined as recurrence or development of new sites of tumor in the pelvis after previous total or partial mesorectal resection for adenocarcinoma of the rectum or distal sigmoid. Curative-intent therapy was considered for patients presenting with resectable local disease (where achieving a R0 resection was deemed feasible). Patients with synchronous oligometastatic disease were deemed candidates for curative treatment. Patients were excluded if they had local excision of the primary tumor only or regrowth after failure of a watch-and-wait strategy, or management of LRRC with a watch-and-wait strategy. Second and third recurrences were excluded from this analysis. The present study was reviewed and approved by the Dutch Medical Research Ethics Committees United–Nieuwegein (registration number: AW24.062/W23.008).

### Neoadjuvant Treatment

All patients undergoing curative-intent therapy for LRRC received neoadjuvant treatment in accordance with the local standard of care at the time. Generally, for radiotherapy-naïve patients with LRRC, full-course CRT was planned consisting of a cumulative dose of 50–50.4 Gy in 25–28 fractions of 1.8–2 Gy. In patients with LRRC with a history of prior radiotherapy, chemo-reirradiation was planned consisting of 30 Gy in 15 fractions of 2 Gy. In both regimens, concurrent oral capecitabine 825 mg/m^2^ was administered on radiotherapy days. Selected patients received ICT prior to CRT, which typically consisted of 3–4 cycles of CAPOX (capecitabine and oxaliplatin), 4–6 cycles of FOLFOX (5-fluorouracil, leucovorin, and oxaliplatin) or 5 cycles of FOLFIRI (5-fluorouracil, leucovorin, and irinotecan). In the early 2000s, the local standard of care was predominantly limited to neoadjuvant CRT alone. During this period, ICT was only administered to patients with an initial palliative intent, often in conjunction with consolidation chemotherapy. From approximately 2010 onwards, administration of neoadjuvant ICT prior to CRT in the curative-intent setting became generally more widely adopted. Moreover, from 2010 onwards, patients underwent baseline pelvic MRI and thoracoabdominal (PET-)CT, with restaging with CT and MRI following completion of neoadjuvant therapy.

### Surgery

Key determinants influencing the surgical approach included anatomical location and degree of involvement of adjacent structures. When indicated, urological or plastic surgery reconstructions were performed. Preliminary intraoperative electron beam radiotherapy (IOERT) indication was assessed within the MDT if resection margins were deemed as “at-risk,” based on mesorectal fascia involvement, extramural vascular invasion, or tumor adherence to adjacent structures on diagnostic MRI. Perioperatively, IOERT indication was re-evaluated by the radiation oncologist and surgical oncologist. Initially, IOERT dosages ranged between 10, 12.5, and 15 Gy in the event of R0, and microscopically (R1) and macroscopically (R2) involved margins. Since 2021, a dose-adapted technique has allowed delivery of a higher surface dose (15.5–16.5 Gy).^12^ Synchronous metastases were usually managed during the interval between neoadjuvant therapy and surgery for LRRC. In a limited number of cases, treatment of synchronous metastases was performed concomitantly with, or following, surgery for LRRC.

Patients received peri- and postoperative care in accordance with local standard of care applicable to the respective time period. While the Enhanced Recovery After Surgery (ERAS) protocol for colorectal cancer care began its developments in the 2000s, no modified ERAS protocol for LRRC was available because of disease heterogeneity. A dedicated ERAS protocol tailored to LRRC was developed at our center and has been widely implemented since 2022. This protocol focuses primarily on prehabilitation, multimodal anesthesia, postoperative early oral intake, and early mobilization programs alongside other key principles aimed at optimizing recovery and improving peri- and postoperative care.^10^

### Follow-Up

Patients were monitored according to the local standard of care applicable during the respective time period. Over time, the intensity and content of follow-up has evolved. This primarily entails incorporation of biannual CT imaging with variable intervals of carcinoembryonic antigen monitoring. The follow-up period extended until the most recent outpatient clinic visit (until July 20, 2026) or until date of death.

### Outcomes

The primary outcome was evaluation of global temporal improvements in LRRC treatment, as demonstrated by changes in (1) overall survival (OS), (2) disease-free survival (DFS), (3) R0 rates, and (4) postoperative complication rates. Cumulative sum (CUSUM) analyses were used to evaluate temporal changes of the abovementioned outcomes over the study period. OS (defined as absence of death) was calculated from the date of LRRC diagnosis until the date of death from any cause or censored at last follow-up. DFS (defined as absence of local re-recurrence or distant metastases) was calculated from the date of LRRC surgery until the date of local re-recurrence or distant metastases, or censored at last follow-up or death. To facilitate clinical interpretation of evolving treatment strategies, patients were also categorized empirically into three defined (approximately equal) subgroups, each representing distinct treatment periods that aligned with major shifts in LRRC management in our center: 2000–2010 (*n* = 170), 2011–2017 (*n* = 208), and 2018–2023 (*n* = 182). The first time period corresponds to the early period of centralization and implementation of neoadjuvant chemo-reirradiation. The second time period corresponds to the growing adoption of emerging curative-intent strategies and implementation of ICT. The third period correlates to the adoption of personalized treatment strategies with all cases routinely discussed in a dedicated LRRC MDT. To ensure comparability across the time periods, we examined statistical differences in treatment outcomes, focusing predominantly on the CUSUM analyses, which highlighted the evolution of care practices over time. Secondary study endpoints were changes in prognostic factors associated with OS and DFS.

### Statistical Analysis

Descriptive statistics were calculated and expressed as mean ± standard deviation or median (interquartile range), depending on parameter distribution. For categorical data, group comparisons were performed using the χ2 test or Fisher’s exact test, as appropriate. Five-year OS and DFS were estimated using the Kaplan–Meier method. Comparisons were made using log rank test. Hazard ratios (HRs) were calculated using Cox proportional hazard analyses. Univariable and multivariable Cox regression survival analyses were performed to identify clinicopathological prognosticators for OS and DFS and adjusted for confounder variables. Covariates were grouped into categorical variables and included in the multivariable model when *p *≤ 0.100. A two-sided *p*-value ≤ 0.05 was considered statistically significant.

CUSUM analyses were used to illustrate dynamic deviations in OS, DFS, R0 rates, and postoperative complication rates, following the methods described by Barrio et al.^13^ The method tracks cumulative changes from an expected performance facilitating identification of shifts over time. Predicted probabilities were calculated using Kaplan–Meier estimates and descriptive statistics for R0 rates and postoperative complication rates. These calculations were used to produce different CUSUM curves and were calculated for OS and DFS. The x-axis represents the year of LRRC therapy, and the y-axis represents the cumulative sum of deviations from the mean. The line ascends for every patient whose OS or DFS deviated positively from the expected survival probability. A CUSUM curve was calculated for R0 resection rate, in which an ascending line reflects a positive deviation from the expected probability. Lastly, a CUSUM curve was calculated for postoperative complication rates, in which an upward trend correlates to an increasing rate of complications beyond the expected threshold, and downward trend suggests reduction in complications. All statistical analyses were performed using IBM SPSS Statistics version 29.0 (IBM Corporation, Armonk, NY, USA).

## Results

### Patient Cohort

**Fig. 1:**
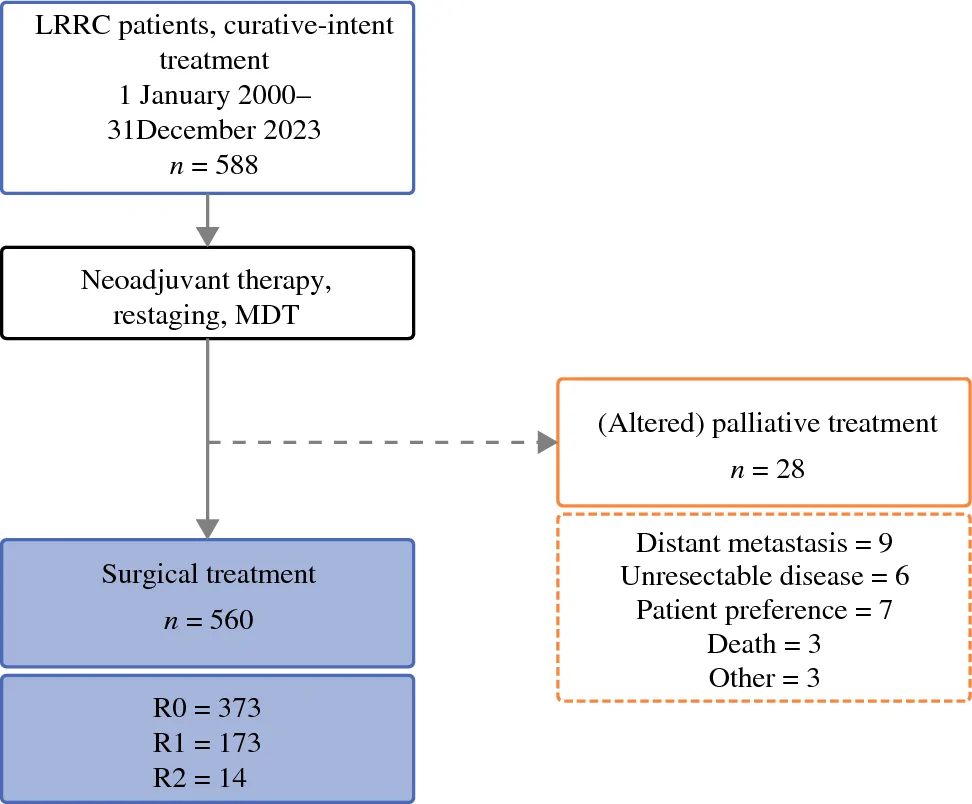
Flow diagram illustrating identification of patients with locally recurrent rectal cancer (LRRC) undergoing curative-intent therapy between 2000 and 2023. MDT, multidisciplinary team; R0, clear resection margin; R1, microscopically involved resection margin; R2, macroscopically involved margins or massive invasion into resection surface

In total, 560 patients with LRRC treated with curative-intent surgical resection between 2000 and 2023 were included (Fig. 1). Baseline, primary, and recurrent tumor characteristics and treatment are presented in Table 1. The median follow-up, measured until death or last follow-up, was 43 (interquartile range 23–73) months.

**Table 1:** Baseline, primary and recurrent tumor characteristics and treatment

Characteristics		Period 1 (2000–2010) *n *= 170	Period 2 (2011–2017) *n *= 208	Period 3 (2018–2023) *n *= 182	*p*-value
Baseline and primary tumor characteristics					
Sex	Male	104 (61)	133 (64)	127 (70)	0.221
	Female	66 (39)	75 (36)	55 (30)	
Age at primary tumor, years		60 (53–67)	61 (53–69)	61 (55–69)	0.128
ASA	I	40 (23)	7 (3)	6 (3)	**<0.001**
	II	98 (58)	164 (79)	142 (78)	
	III	17 (10)	37 (18)	34 (19)	
	IV	1 (1)	-	-	
	Unknown	14 (8)	-	-	
Subsite	Rectum	156 (92)	179 (86)	157 (86)	0.406
	Sigmoid	11 (6)	25 (12)	20 (11)	
	Rectosigmoid	3 (2)	4 (2)	5 (3)	
Pathological tumor category, primary	pT0–2	29 (17)	48 (23)	45 (25)	0.207
	pT3	119 (70)	129 (62)	106 (58)	
	pT4	18 (11)	28 (13)	29 (16)	
	Unknown	4 (2)	3 (2)	2 (1)	
Pathological nodal category, primary	pN0	84 (50)	86 (41)	98 (54)	0.093
	pN1+	82 (48)	119 (57)	80 (44)	
	Unknown	4 (2)	3 (2)	4 (2)	
Neoadjuvant therapy, primary	(Chemo)radiotherapy	110 (65)	130 (63)	106 (58)	**0.007**
	Induction chemotherapy	1 (1)	12 (6)	22 (12)	**<0.001**
Surgery primary	Low anterior resection	94 (55)	110 (53)	104 (57)	0.822
	Abdominoperineal resection	54 (32)	62 (30)	52 (29)	
	Sigmoid resection	16 (9)	22 (10)	19 (10)	
	Hartmann	5 (3)	11 (5)	4 (2)	
	Exenterative surgery	1 (1)	3 (2)	3 (2)	
Adjuvant therapy	Chemotherapy	26 (15)	50 (24)	23 (13)	**<0.001**
	Radiotherapy	3 (2)	1 (1)	2 (1)	
Characteristics recurrent tumor and treatment					
Recurrence interval	Median, months (IQR)	33 (19-49)	26 (17-43)	29 (19-52)	**0.029**
Multifocality	Yes	14 (8)	40 (19)	40 (22)	**<0.001**
	No	143 (84)	168 (81)	142 (78)	
	Unknown	13 (8)	-	-	
Synchronous metastases	Yes	9 (5)	36 (17)	38 (21)	**<0.001**
	No	161 (95)	172 (83)	144 (79)	
Neoadjuvant therapy, recurrence	Chemo-(re)irradiation	148 (87)	172 (83)	170 (93)	**0.006**
	Induction chemotherapy	12 (7)	95 (46)	107 (59)	**<0.001**
Surgery, recurrence	Low anterior resection	33 (20)	43 (21)	21 (12)	**0.003**
	Abdominoperineal resection	82 (48)	84 (40)	65 (36)	
	Exenterative surgery	24 (14)	27 (13)	55 (30)	
	Debulking	31 (18)	46 (22)	37 (20)	
	Resection n.o.s	-	8 (4)	4 (2)	
IOERT, recurrence	Yes	153 (90)	153 (74)	133 (73)	**<0.001**
Pathological resection margin, recurrence	R0	101 (59)	130 (63)	142 (78)	**<0.001**
	R1	58 (34)	75 (36)	40 (22)	
	R2	11 (7)	3 (1)	-	
Pathological response	pCR	-	13 (6)	20 (11)	**<0.001**

Data are presented as n (%) or median (interquartile range) unless otherwise indicated.

ASA, American Society of Anesthesiologists Physical Status Classification System; IORT, intraoperative radiotherapy; NOS, not otherwise specified; pCR, pathological complete response.; pN, pathological nodal stage; pT, pathological tumor stage; R0, clear resection margin; R1, microscopically involved margin; R2, macroscopically involved margins or massive invasion into resection surface.

Bold values denote statistical significance

#### Changes in Baseline Characteristics

In the more recent periods, patients presented with higher American Society of Anesthesiologists Physical Status Classification System (ASA) scores (ASA I 23%, 3%, 3%; ASA II 58%, 79%, 78%; ASA III 10%, 18%, 19%, in periods 1, 2, and 3, respectively, *p *< 0.001), indicating greater preoperative physical burden. Additionally, in the more recent periods, a larger proportion of patients were diagnosed with multifocal disease (8%, 19%, 22%, in periods 1, 2, and 3, respectively,* p *< 0.001) and synchronous metastasis (5%, 17%, 21%, in periods 1, 2, and 3, respectively, *p *< 0.001).

#### Changes in Neoadjuvant Therapy and Surgery

**Fig. 2:**
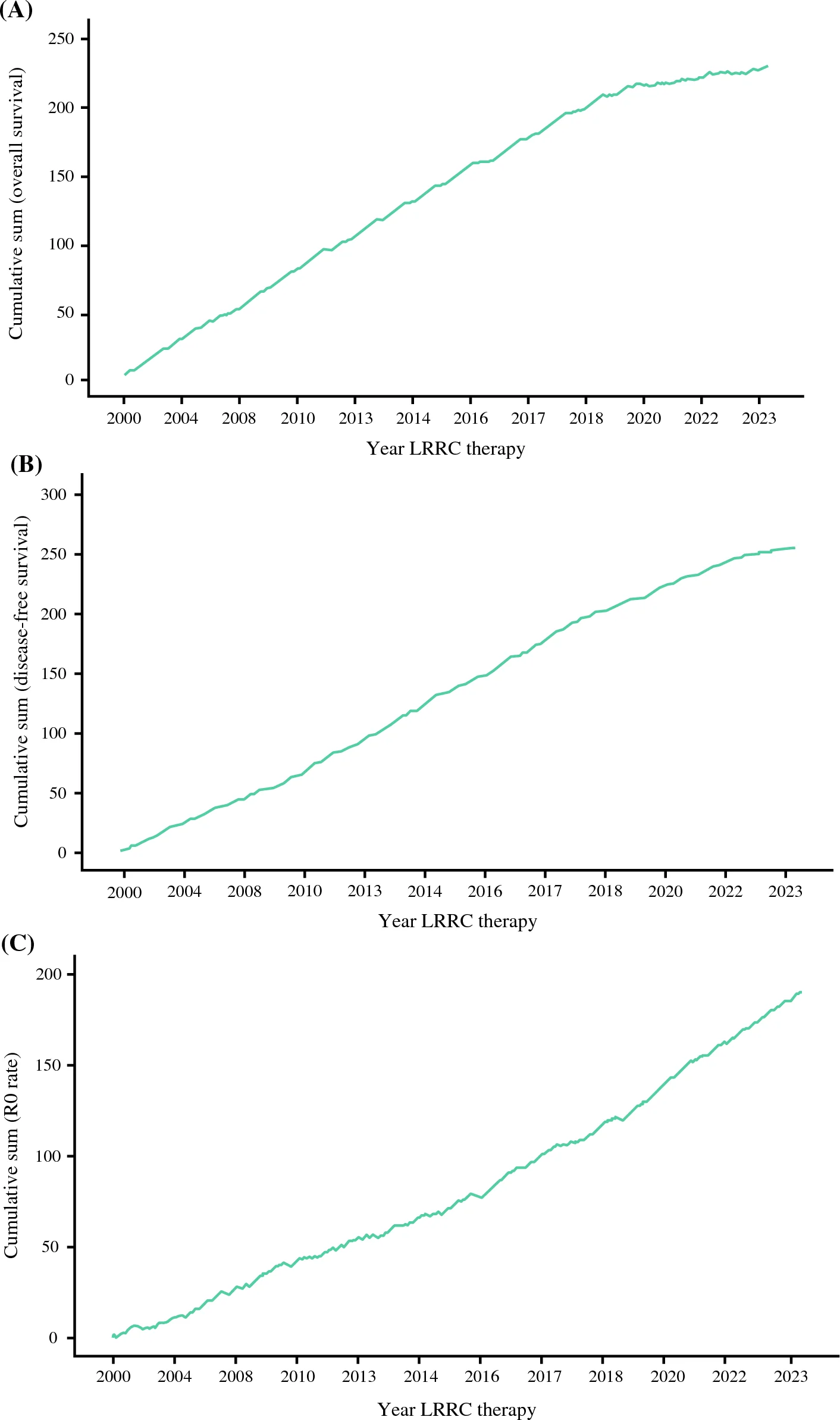

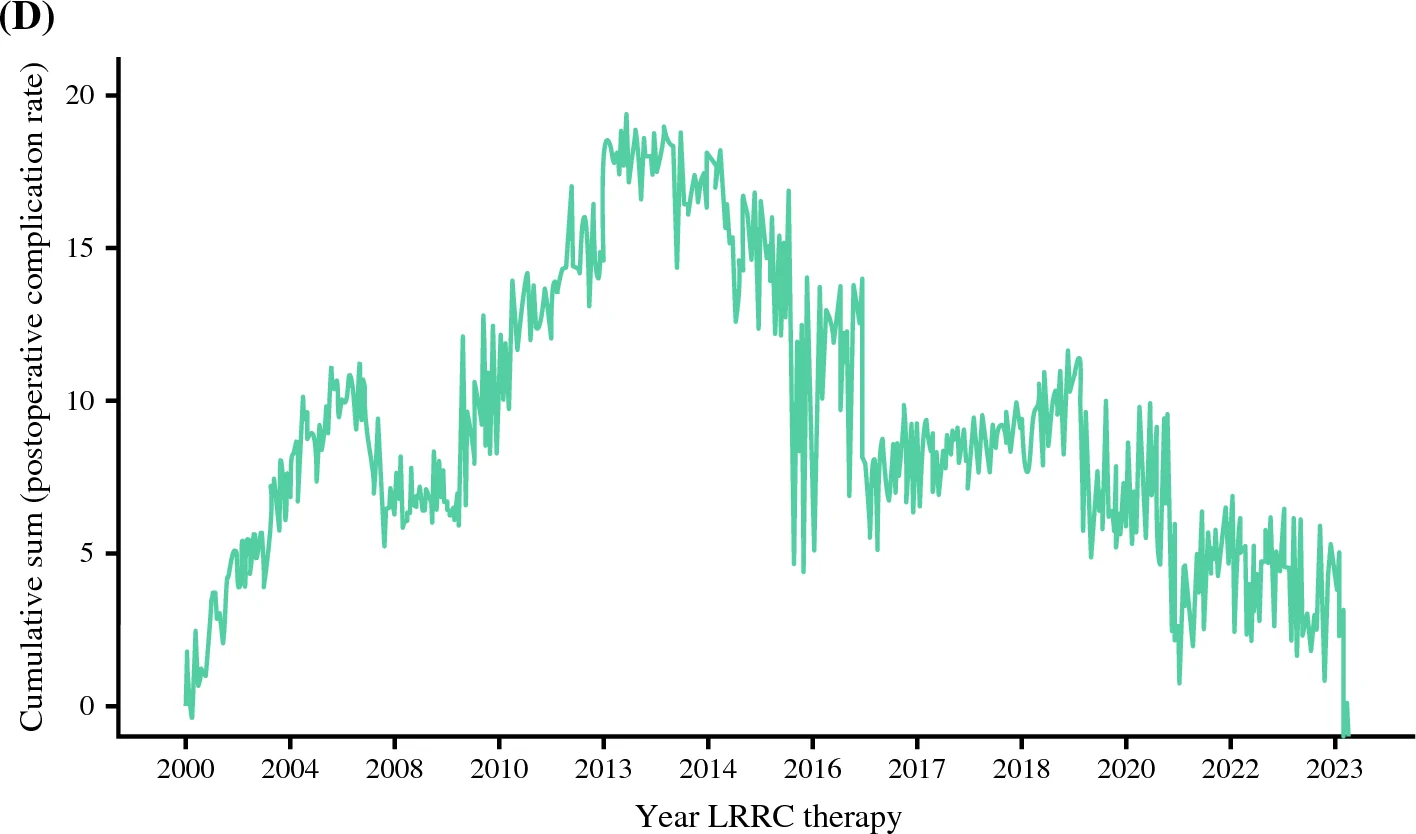
CUSUM curves. **A** Overall survival, **B** disease-free survival, **C** R0 resection rate, and **D** complication rates according to year of locally recurrent rectal cancer (LRRC) therapy. Note: Spacing between years on the x-axis is uniform and does not correspond to the elapsed time between years

Neoadjuvant ICT has increasingly been implemented over time (7%, 46%, 59%, in periods 1, 2, and 3, respectively, *p *< 0.001). An increasing proportion of patients underwent exenterative surgery (14%, 13%, 30%, in periods 1, 2, and 3, respectively, *p* = 0.003), whereas IOERT use decreased (90%, 74%, 73%, in periods 1, 2, and 3, respectively, *p *< 0.001). R0 rates improved significantly over time (59%, 63%, 78%, in periods 1, 2, and 3, respectively, *p *< 0.001). The CUSUM curve for R0 resection rates illustrates an upward trend over time, demonstrating a cumulative improvement (Fig. 2C).

### Trends in Outcomes

#### Changes in Oncological Outcomes

The CUSUM curve for OS (Fig. 2A) illustrates an upward trend over time, indicating a cumulative improvement in survival relative to the reference survival probability. A similar upward trend was observed for DFS (Fig. 2B).

**Fig. 3:**
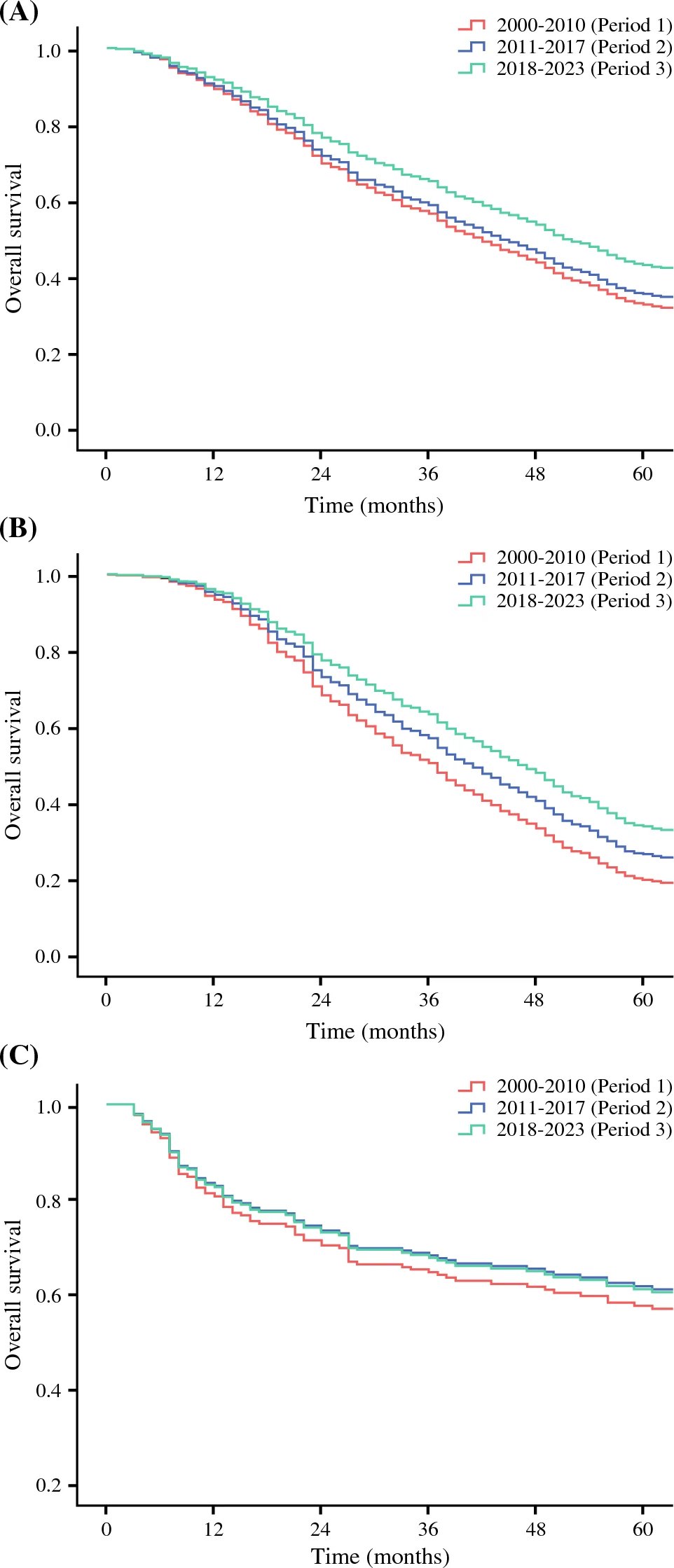
Cox proportional hazards model for **A** overall survival and **B** overall survival after diagnosis of relapsing disease (local re-recurrence or distant metastasis), **C** overall survival for patients who did not develop relapsing disease (local re-recurrence or distant metastasis). **A** *p *= 0.524 (period 2 vs. 1, hazard ratio [HR] 0.93; 95% confidence interval [CI] 0.75–1.16), *p *= 0.030 (period 3 vs. 1, HR 0.75; 95% CI 0.58–0.97). **B** *p *= 0.121 (period 2 vs 1, HR 0.82; 95% CI 0.64–1.05), *p *= 0.008 (period 3 vs. 1, HR 0.67; 95% CI 0.50–0.90). **C** *p *= 0.605 (period 2 vs. 1, HR 0.88; 95% CI 0.53–1.45), *p *= 0.667 (period 3 vs 1, HR 0.89; 95% CI 0.54–1.49)

In the univariable Cox regression analyses, successive treatment periods were identified as a significant prognosticator associated with OS (Fig. 3A). The most recent period, period 3, was associated with improved survival (HR 0.75; 95% confidence interval [CI] 0.58–0.97; *p* = 0.030) compared with period 1. The 5-year OS was 31.2% for patients treated in period 1 versus 35.1% in period 2 (*p* = 0.415) versus 43.6% in period 3 (*p* = 0.017) (Supplementary Figure S1). In subgroup analyses, patients diagnosed with recurrent disease (defined as local re-recurrence or distant metastasis after curative treatment for LRRC) demonstrated improved OS in period 3 compared with period 1 (HR 0.67; 95% CI 0.50–0.90, *p* = 0.008, Fig. 3B). Conversely, for patients who did not develop recurrent disease in follow-up, OS was comparable across all treatment time periods (Fig. 3C).

#### Changes in Surgical Morbidity and Mortality

The CUSUM curve demonstrated an upward trend until approximately 2013, indicating an increase in complication rates (Fig. 2D). From 2013 onwards, a marked downwards curve is observed, indicating reduction in complication rates. Postoperative complications and 1-year mortality rates are presented in Table 2. Successive treatment periods illustrate a decreasing rate in overall postoperative complications (*p* = 0.027). A non-significant trend of decreasing 1-year mortality rate was observed (*p* = 0.125).

**Table 2:** Postoperative morbidity and mortality rates over time.

Morbidity and mortality		Period 1 (2000–2010) *n=170*	Period 2 (2011–2017) *n=208*	Period 3 (2018–2023) *n=182*	*p-Value*
Postoperative complications, Clavien–Dindo	Grade 0	41 (24)	66 (31)	68 (37)	**0.027**
	Grade I–II	48 (28)	85 (41)	65 (36)	
	Grade IIIa–IIIb	73 (43)	56 (27)	38 (21)	
	Grade IV	7 (4)	1 (1)	10 (5)	
	Grade V	1 (1)	-	1	
1-year mortality	N (%)	24 (14)	18 (9)	15 (8)	0.125

Bold values denote statistical significance

### Trends in Prognostic Factors

Univariable and multivariable Cox regression survival analyses are presented in Tables S1–S3. Notably, R1/R2 resection was the only factor associated with worse OS in all three periods in the multivariable analyses (period 1 HR 1.75; 95% CI 1.23–2.47, period 2 HR 2.62; 95% CI 1.87–3.67, period 3 HR 2.60; 95% CI 1.65–4.07). Additional factors significantly associated with worse OS were primary pN1+ nodal status (period 1 HR 1.55; 95% CI 1.11–2.18) and multifocal disease (period 2 HR 1.56; 95% CI 1.06–2.30). No collinearity was observed among the variables included in the analyses.

## Discussion

The current study aimed to evaluate developments in LRRC treatment over an extended period of time, focusing on cumulative changes and trends in oncological outcomes. Analyses revealed that survival-related outcomes for curatively treated patients with LRRC have steadily improved over the past two decades. These improvements were accompanied by increasing R0 resection rates despite a progressively more complex patient population, characterized by higher rates of preoperative physical burden, multifocal disease, and synchronous metastases. Collectively, these findings may reflect advances in diagnostics, neoadjuvant therapy, peri- and postoperative care, and centralization of care. CUSUM analyses illustrated a gradual and sustained improvement in these outcomes over time.

Accurate locoregional assessment is key for patients diagnosed with LRRC. According to the Dutch national guidelines, LRRC diagnosis should be made clinically based on a suspicious or growing lesion on MRI combined with a suggestive (PET-)CT or rising carcinoembryonic antigen and MDT consensus.^5^ A diagnostic thoracoabdominal CT should be performed to assess the presence of distant metastases. These imaging modalities play a crucial role in evaluating tumor location, involvement of adjacent structures and assessment of (potential) resectability, thereby informing treatment intent. Although both MRI and CT have been available since the early 2000s, their routine use for LRRC locoregional assessment became more widely integrated into clinical practice during the later years of the study period. Advances in MRI technology have enabled higher spatial resolution, improved image quality, and shorter acquisition times. T2-weighted MRI, for instance, has a high accuracy (95%), sensitivity (80–91%), and specificity (86–100%) in LRRC.^14,15^ Challenges in differentiating between postoperative changes, fibrosis, and tumor remain, with the positive predictive value for identifying tumor invasion into surrounding structures varying widely (53–100%).^16,17^ The addition of diffusion-weighted imaging has further enhanced the ability to distinguish fibrosis from tumour.^18–22^ Together with increased centralization in tertiary referral centers and the establishment of expert MDTs,^23,24^ these developments have likely improved diagnostic confidence and informed treatment planning throughout the study period. Other changes in LRRC management, including evolving surgical practice and broader implementation of perioperative optimization programs such as ERAS,^10^ as well as improvements in perioperative care, may also have contributed to the reductions in postoperative complications and 1-year postoperative mortality and, ultimately, to the improved OS observed over time. These developments may also have enabled curative-intent treatment in patients with a greater preoperative comorbidity burden, as reflected by the increasing proportion of patients with higher ASA classifications over time. Lastly, the improved post-recurrence survival observed in more recent treatment periods suggests that advances in oncological management after recurrence, including palliative systemic therapy, may also have contributed to the observed improvements in OS. Taken together, these developments reflect the evolving multidisciplinary management of LRRC over the study period, although their individual contributions cannot be determined from the present study.

During the study period, various new neoadjuvant and surgical treatment options were implemented. Implementation of neoadjuvant treatment for LRRC has significantly increased, with broad application of CRT and an increasing number of patients receiving ICT. Although surgical resection was once considered the cornerstone of treatment, the paradigm is now shifting towards a more biology-driven approach, prioritizing neoadjuvant therapies to induce preoperative downstaging and to increase the chances of an R0 resection and possibly improve oncological outcomes.^4^ Chemo-(re)irradiation has been used routinely for some time in patients with LRRC^25,26^, whereas ICT is not yet routinely administered. In the current study, increasing numbers of patients received ICT, in part within the context of the PelvEx II trial. Various retrospective cohorts have demonstrated that the addition of ICT may increase the likelihood of R0 resection and pathologic complete response (pCR), which are both prognostic factors.^27–29^ In a comparative analysis, the addition of ICT led to less extensive resection than CRT only, although no significant differences in oncological outcomes were observed.^28^ Based on the currently available data, it is unclear whether it is the addition of ICT or factors such as patient selection that result in favorable outcomes. Data from the ongoing PelvEx II trial will provide more insight into the added value of ICT.^8^

Our study demonstrates a notable increase in the R0 resection rate, rising from approximately 60% in the early 2000s to nearly 80% in more recent years. This improvement might be explained by advances in neoadjuvant treatment and surgical techniques; however, it may also reflect more precise patient selection enabled by enhanced preoperative diagnostics. The aforementioned advances of imaging modalities have likely improved locoregional restaging, which facilitates better-informed decisions regarding the selection of patients for salvage surgery. However, it is debatable whether achieving an R0 resection is prognostic for the oncological outcome or indicative of the tumor biology, or both. Recent developments in LRRC research emphasize the importance of evaluating the biological behavior of tumors before, during, and after treatment, as it might be the most important factor determining the outcome.^30–33^ Previous reports show that specific clinical, radiological, and pathological features can be used to make an estimation of the favorability of biological behavior, ranging from slowly progressive, relatively favorable tumors to aggressively expanding, unfavorable tumours.^2,5^

The main limitation of this study is its retrospective nature, resulting in potential selection bias. There is no complete data on the proportion of patients undergoing palliative treatment at baseline, as most are referred back to their referring hospital once palliative treatment is decided upon, and some patients may have never been referred because of the extent of the disease. Data on patients who presented with disease progression at restaging in neoadjuvant setting are limited, precluding assessment of the potential selection effect of neoadjuvant treatment. The multifactorial nature of changes over the study period renders it difficult to highlight the influence of one specific prognostic factor on outcome. Nevertheless, the population size, length of follow-up, and the completeness of the data allow for important insights into the development of LRRC treatment over the past two decades.

## Conclusions

Oncological outcomes for patients with LRRC treated with curative intent have steadily improved over the past two decades. The cumulative effect of various improvements in diagnostics, neoadjuvant treatment, peri- and postoperative care, and centralization of care is likely to have contributed to these trends.

## Supplementary Information

Below is the link to the electronic supplementary material.

Supplementary file 1 (DOCX 113 kb)

## Data Availability

Data supporting this study’s findings are available on request from the corresponding author.

## Abbreviations

CRT: Chemoradiotherapy
CT: Computed tomography
CUSUM: Cumulative sum
DFS: Disease-free survival
DWI: Diffusion-weighted imaging
ERAS: Enhanced recovery after surgery
ICT: Induction chemotherapy
IOERT: Intraoperative electron beam radiotherapy
IQR: Interquartile ranges
LRRC: Locally recurrent rectal cancer
LRFS: Local re-recurrence-free survival
MDT: Multidisciplinary team
MRI: Magnetic resonance imaging
OS: Overall survival
PET: Positron emission tomography
R0: Clear resection margin
TME: Total mesorectal excision
