# Supplementary material for: Evolution of Prognosis and Management in Curative Treatment of Locally Recurrent Rectal Cancer: A Comprehensive Analysis from 2000 to 2023
Source: Ann Surg Oncol. 2026 Aug 20;33(12):10616–26. doi: 10.1245/s10434-026-20398-6 (PMC13675363; doi:10.1245/s10434-026-20398-6)
Supplement: Supplementary file 1 [file 10434_2026_20398_MOESM1_ESM.docx]

**Tables and figures**

**Figure S1.** Kaplan-Meier curve for overall survival, according to treatment era. Pairwise comparisons:
*P* = 0.017 (period 1 versus 3), *P* = 0.129 (period 2 versus 3), *P* = 0.415 (period 1 versus 2).

**Table S1.** Univariable and multivariable analyses for risk factors for overall and disease-free survival in period 1 (2000-2010, n=170)

**Table S2.** Univariable and multivariable analyses for risk factors for overall and disease-free survival in period 2 (2010-2017, n=208)

**Table S3.** Univariable and multivariable analyses for risk factors for overall and disease-free survival in period 3 (2018-2023, n=182)

**Fig. S1.** Kaplan-Meier curve for overall survival, according to treatment era. Pairwise comparisons:
*P* = 0.017 (period 1 versus 3), *P* = 0.129 (period 2 versus 3), *P* = 0.519 (period 1 versus 2).

No. at risk (events):
Period 1	170 (0)	146 (24)	110 (60)	85 (85)	68 (102)	53 (117)
Period 2	208 (0)	192 (16)	158 (50)	133 (75)	100 (108)	74 (134)
Period 3	182 (0)	168 (14)	139 (43)	116 (64)	81 (81)	56 (95)

**Table S1.** Univariable and multivariable analyses for risk factors for overall and disease-free survival in period 1 (2000-2010, n=170)

|  | Overall survival | | | | Disease-free survival | | | |
| --- | --- | --- | --- | --- | --- | --- | --- | --- |
|  | Univariable HR (95% CI) | *p* | Multivariable HR (95% CI) | *p* | Univariable HR (95% CI) | *p* | Multivariable HR (95% CI) | *p* |
| **Age** | 1.03 (1.01-1.05) | **<0.001** |  | **<0.001** |  | 0.454 |  |  |
|  |  |  | 1.02 (1.02-1.05) |  | 1.01 (0.99-1.02) |  |  |  |
| **Gender**  **Male** (104)  **Female** (66) | 1.00  1.00 (0.72-1.38) | 0.978 |  |  | 1.00  0.99 (0.68-1.44) | 0.943 |  |  |
| **ASA**  **I-II** (138)  **III-IV** (18) | 1.00  0.75 (0.44-1.26) | 0.272 |  |  | 1.00  0.53 (0.26-1.09) | **0.085** | 1.00  0.59 (0.28-1.22) | 0.155 |
| **Pathological tumour category, primary**  **pT0-2** (29)  **pT3** (119)  **pT4** (18) | 1.00  1.25 (0.81-1.92)  1.16 (0.61-2.18) | 0.597 |  |  | 1.00  1.41 (0.84-2.37)  1.37 (0.63-2.99) | 0.437 |  |  |
| **Pathological nodal category, primary**  **pN0** (84)  **pN1+** (82) | 1.00  1.65 (1.18-2.29) | **0.003** |  | **0.010** |  | **0.004** |  | **0.042** |
|  |  |  | 1.00 |  | 1.00 |  | 1.00 |  |
|  |  |  | 1.55 (1.11-2.18) |  | 1.75 (1.20-2.54) |  | 1.50 (1.01-2.22) |  |
| **Neoadjuvant therapy, primary**  **None** (54)  **CRT only** (109) | 1.00  1.64 (1.14-2.34) | **0.027** | 1.00  1.51 (1.05-2.18) | **0.027** | 1.00 1.28 (0.86-1.92) | 0.477 |  |  |
| **Surgery, primary**  **Sigmoid resection/Hartmann** (21)  **LAR** (94)  **APR** (54) | 1.00  1.31 (0.77-2.24)  1.48 (0.84-2.61) | 0.410 |  |  | 1.00  1.11 (0.61-2.03)  1.27 (0.68-2.39) | 0.321 |  |  |
| **Adjuvant therapy, primary**  **No** (141)  **Yes** (29) | 1.00  0.81 (0.52-1.25) | 0.342 |  |  | 1.00  1.21 (0.77-1.91) | 0.416 |  |  |
| **Multifocality, recurrence**  **No** (143)  **Yes** (14) | 1.00  1.57 (0.89-2.78) | 0.125 |  |  | 1.00  1.71 (0.91-3.19) | **0.095** | 1.00  1.45 (0.71-2.97) | 0.312 |
| **Neoadjuvant induction chemotherapy, recurrence**  **No** (158)  **Yes** (12) | 1.00  1.03 (0.54-1.96) | 0.926 |  |  | 1.00  1.35 (0.69-2.67) | 0.385 |  |  |
| **Neoadjuvant (chemo)radiotherapy, recurrence**  **Yes** (148)  **No** (22) | 1.00  1.83 (0.51-1.34) | 0.438 |  |  | 1.00  1.23 (0.70-2.15) | 0.475 |  |  |
| **Surgery, recurrence**  **LAR** (33)  **APR** (82)  **Exenterative surgery/debulking** (55) | 1.00  0.98 (0.63-1.53)  1.16 (0.73-1.86) | 0.648 |  |  | 1.00  0.86 (0.51-1.44)  1.28 (0.75-2.18) | 0.169 |  |  |
| **IOERT, recurrence**  **Yes** (153)  **No** (17) | 1.00  0.95 (0.55-1.61) | 0.837 |  |  | 1.00  0.98 (0.55-1.75) | 0.949 |  |  |
| **Resection margin**  **R0** (101)  **R1/R2** (69) | 1.00  1.66 (1.19-2.31) | **0.003** | 1.00  1.75 (1.23-2.47) | **0.002** | 1.00  1.92 (1.32-2.80) | **<0.001** | 1.00  1.84 (1.22-2.75) | **0.003** |
| **Synchronous metastasis, recurrence**  **No** (161)  **Yes** (9) | 1.00  0.95 (0.47-1.94) | 0.889 |  |  | 1.00  2.14 (1.04-4.41) | **0.039** | 1.00  2.10 (0.94-4.65) | 0.069 |
| **Year surgery, recurrence**  Continuous | 0.95 (0.90-0.99) | **0.028** | 0.95 (0.91-1.01) | 0.086 | 1.02 (0.95-1.08) | 0.516 |  |  |

*CRT* = (chemo)radiotherapy; *ICT* = induction chemotherapy; *LAR* = low anterior resection; *APR* = abdominoperineal resection; *IOERT* = intraoperative radiotherapy; *R0* = radical resection; *R1* = irradical resection; *R2* = macroscopically involved margins or massive invasion into resection surface.

**Table S2.** Univariable and multivariable analyses for risk factors for overall and disease-free survival in period 2 (2010-2017, n=208)

|  | Overall survival | | | | Disease-free survival | | | |
| --- | --- | --- | --- | --- | --- | --- | --- | --- |
|  | Univariable HR (95% CI) | *p* | Multivariable HR (95% CI) | *p* | Univariable HR (95% CI) | *p* | Multivariable HR (95% CI) | *p* |
| **Age** | 1.02 (1.00-1.04) | **0.011** |  | **<0.001** |  | 0.258 |  |  |
|  |  |  | 1.03 (0.02-1.05) |  | 1.00 (0.98-1.01) |  |  |  |
| **Gender**  **Male** (133)  **Female** (75) | 1.00  0.84 (0.62-1.15) | 0.288 |  |  | 1.00  0.83 (0.60-1.15) | 0.265 |  |  |
| **ASA**  **I-II** (171)  **III-IV** (37) | 1.00  0.94 (0.64-1.38) | 0.746 |  |  | 1.00  0.85 (0.56-1.29) | 0.436 |  |  |
| **Pathological tumour category, primary**  **pT0-2** (48)  **pT3** (129)  **pT4** (28) | 1.00  1.02 (0.72-1.46)  1.33 (0.81-2.20) | 0.464 |  |  | 1.00  1.01 (0.69-1.49)  1.87 (1.09-3.20) | **0.030** | 1.00  0.94 (0.63-1.40)  1.74 (0.94-3.24) | 0.074 |
| **Pathological nodal category, primary**  **pN0** (86)  **pN1+** (119) | 1.00  1.11 (0.82-1.50) | 0.518 |  |  |  | 0.128 |  |  |
|  |  |  |  |  | 1.00 |  |  |  |
|  |  |  |  |  | 1.29 (0.93-1.78) |  |  |  |
| **Neoadjuvant therapy, primary**  **None** (75)  **ICT + CRT** (9)  **CRT only** (121) | 1.00  1.42 (1.04-1.95)  1.34 (0.64-2.80) | **0.092** | 1.00  1.39 (0.93-2.09)  0.91 (0.39-2.12) | 0.178 | 1.00  1.55 (1.10-2.17)  1.59 (0.76-3.36) | **0.036** | 1.00 1.54 (0.97-2.49)  0.81 (0.34-1.94) | 0.079 |
| **Surgery, primary**  **Sigmoid resection/Hartmann** (33)  **LAR** (110)  **APR** (62) | 1.00  1.17 (0.76-1.78)  1.28 (0.81-2.02) | 0.435 |  |  | 1.00  1.60 (1.00-2.58)  1.62 (0.96-2.72) | 0.115 |  |  |
| **Adjuvant therapy, primary**  **No** (157)  **Yes** (51) | 1.00  1.07 (0.76-1.52) | 0.682 |  |  | 1.00  1.01 (0.70-1.45) | 0.954 |  |  |
| **Multifocality, recurrence**  **No** (168)  **Yes** (40) | 1.00  1.40 (0.97-2.03) | **0.075** | 1.00  1.56 (1.06-2.30) | **0.025** | 1.00  1.63 (1.11-2.39) | **0.014** | 1.00  1.61 (1.02-2.42) | **0.022** |
| **Neoadjuvant induction chemotherapy, recurrence**  **No** (113)  **Yes** (95) | 1.00  0.97 (0.72-1.23) | 0.814 |  |  | 1.00  1.06 (0.77-1.45) | 0.732 |  |  |
| **Neoadjuvant (chemo)radiotherapy, recurrence**  **Yes** (172)  **No** (36) | 1.00  0.86 (0.65-1.42) | 0.833 |  |  | 1.00  0.85 (0.55-1.30) | 0.446 |  |  |
| **Surgery, recurrence**  **LAR** (43)  **APR** (84)  **Exenterative surgery/debulking** (73) | 1.00  0.97 (0.65-1.46)  1.69 (1.12-2.55) | **0.003** | 1.00  0.80 (0.48-1.31)  1.32 (0.89-2.19) | **0.019** | 1.00  1.40 (0.88-2.21)  2.09 (1.31-3.34) | **0.005** | 1.00  1.04 (0.60-1.82)  1.23 (0.69-2.19) | 0.651 |
| **IOERT, recurrence**  **Yes** (153)  **No** (55) | 1.00  0.94 (0.67-1.32) | 0.712 |  |  | 1.00  0.92 (0.64-1.31) | 0.629 |  |  |
| **Resection margin**  **R0** (130)  **R1/R2** (78) | 1.00  2.33 (1.71-3.18) | **<0.001** | 1.00  2.62 (1.87-3.67) | **<0.001** | 1.00  2.19 (1.58-3.03) | **<0.001** | 1.00  1.83 (1.29-2.60) | **<0.001** |
| **Synchronous metastasis, recurrence**  **No** (172)  **Yes** (36) | 1.00  1.26 (0.86-1.85) | 0.245 |  |  | 1.00  1.96 (1.33-2.91) | **<0.001** | 1.00  1.74 (1.11-2.73) | **0.016** |
| **Year surgery, recurrence**  Continuous | 0.97 (0.89-1.04) | 0.360 |  |  | 1.02 (0.95-1.12) | 0.562 |  |  |

*CRT* = (chemo)radiotherapy; *ICT* = induction chemotherapy; *LAR* = low anterior resection; *APR* = abdominoperineal resection; *IOERT* = intraoperative radiotherapy; *R0* = radical resection; *R1* = irradical resection; *R2* = macroscopically involved margins or massive invasion into resection surface.

**Table S3.** Univariable and multivariable analyses for risk factors for overall and disease-free survival in period 3 (2018-2023, n=182)

|  | Overall survival | | | | Disease-free survival | | | |
| --- | --- | --- | --- | --- | --- | --- | --- | --- |
|  | Univariable HR (95% CI) | *p* | Multivariable HR (95% CI) | *p* | Univariable HR (95% CI) | *p* | Multivariable HR (95% CI) | *p* |
| **Age** | 1.01 (0.99-1.03) | 0.546 |  |  |  | 0.656 |  |  |
|  |  |  |  |  | 1.00 (0.97-1.02) |  |  |  |
| **Gender**  **Male** (127)  **Female** (55) | 1.00  0.95 (0.63-1.45) | 0.822 |  |  | 1.00  1.53 (1.01-2.30) | **0.044** | 1.00  1.34 (0.87-2.05) | 0.185 |
| **ASA**  **I-II** (148)  **III-IV** (34) | 1.00  1.27 (0.80-2.04) | 0.312 |  |  | 1.00  0.86 (0.48-1.54) | 0.611 |  |  |
| **Pathological tumour category, primary**  **pT0-2** (45)  **pT3** (106)  **pT4** (29) | 1.00  1.38 (0.85-2.25)  1.40 (0.74-2.65) | 0.411 |  |  | 1.00  1.50 (0.89-2.52)  2.07 (1.05-4.07) | 0.104 |  |  |
| **Pathological nodal category, primary**  **pN0** (98)  **pN1+** (80) | 1.00  1.06 (0.72-1.55) | 0.784 |  |  |  | **0.064** |  | 0.217 |
|  |  |  |  |  |  |  |  |  |
|  |  |  |  |  | 1.00  1.45 (0.98-2.13) |  | 1.00  1.29 (0.86-1.92) |  |
| **Neoadjuvant therapy, primary**  **None** (74)  **ICT + CRT** (20)  **CRT only** (86) | 1.00  1.48 (0.99-2.23)  1.31 (0.67-2.57) | 0.168 |  |  | 1.00  1.09 (0.72-1.64)  1.12 (0.57-2.17) | 0.905 |  |  |
| **Surgery, primary**  **Sigmoid resection/Hartmann** (4)  **LAR** (104)  **APR** (52) | 1.00  1.38 (0.73-2.62)  1.39 (0.70-2.77) | 0.181 |  |  | 1.00  0.73 (0.41-1.31)  0.74 (0.39-1.41) | 0.757 |  |  |
| **Adjuvant therapy, primary**  **No** (157)  **Yes** (25) | 1.00  1.52 (0.91-2.52) | 0.108 |  |  | 1.00  1.09 (0.65-1.85) | 0.737 |  |  |
| **Multifocality, recurrence**  **No** (142)  **Yes** (40) | 1.00  1.50 (0.98-2.30) | **0.061** | 1.00  1.48 (0.95-2.30) | 0.084 | 1.00  1.26 (0.82-1.94) | 0.289 |  |  |
| **Neoadjuvant induction chemotherapy, recurrence**  **No** (75)  **Yes** (107) | 1.00  0.85 (0.58-1.26) | 0.430 |  |  | 1.00  0.87 (0.58-1.30) | 0.492 |  |  |
| **Neoadjuvant (chemo)radiotherapy, recurrence**  **Yes** (170)  **No** (12) | 1.00  1.35 (0.68-2.67) | 0.391 |  |  | 1.00  1.52 (0.76-3.01) | 0.234 |  |  |
| **Surgery, recurrence**  **LAR** (21)  **APR** (65)  **Exenterative surgery/debulking** (92) | 1.00  1.02 (0.51-2.01)  1.66 (0.87-3.17) | **0.046** | 1.00  0.99 (0.44-1.77)  1.57 (0.82-3.01) | **0.025** | 1.00  0.78 (0.42-1.44)  0.89 (0.49-1.61) | 0.696 |  |  |
| **IOERT, recurrence**  **Yes** (133)  **No** (49) | 1.00  1.15 (0.76-1.73) | 0.510 |  |  | 1.00  1.27 (0.84-1.93) | 0.258 |  |  |
| **Resection margin**  **R0** (142)  **R1/R2** (40) | 1.00  2.93 (1.94-4.44) | **<0.001** | 1.00  2.60 (1.65-4.07) | **<0.001** | 1.00  2.34 (1.52-3.62) | **<0.001** | 1.00  1.80 (1.13-2.87) | **0.014** |
| **Synchronous metastasis, recurrence**  **No** (144)  **Yes** (38) | 1.00  2.28 (1.51-3.45) | **<0.001** | 1.00  1.75 (1.13-2.72) | **0.013** | 1.00  2.70 (1.73-4.22) | **<0.001** | 1.00  2.20 (1.36-3.53) | **0.001** |
| **Year surgery, recurrence**  Continuous | 0.92 (0.82-1.04) | 0.185 |  |  | 1.02 (0.92-1.14) | 0.713 |  |  |

*CRT* = (chemo)radiotherapy; *ICT* = induction chemotherapy; *LAR* = low anterior resection; *APR* = abdominoperineal resection; *IOERT* = intraoperative radiotherapy; *R0* = radical resection; *R1* = irradical resection; *R2* = macroscopically involved margins or massive invasion into resection surface.
